# Biphenyl Phytoalexin in *Sorbus pohuashanensis* Suspension Cell Induced by Yeast Extract

**DOI:** 10.3390/molecules21091180

**Published:** 2016-09-14

**Authors:** Liangyun Zhou, Jian Yang, Guang Yang, Chuanzhi Kang, Wenjuan Xiao, Chaogeng Lv, Sheng Wang, Jinfu Tang, Lanping Guo

**Affiliations:** 1 The State Key Laboratory Breeding Base of Dao-di Herbs, National Resource Center for Chinese Materia Medica, China Academy of Chinese Medical Sciences, Beijing 100700, China; zhouliangyun0501@163.com (L.Z.); yangchem2012@163.com (J.Y.); hbykdxyg2008@163.com (G.Y.); kangchuanzhi1103@163.com (C.K.); lcgfim@126.com (C.L.); mmcniu@163.com (S.W.); jinfutang@126.com (J.T.); 2 Pixian Chinese Medicine Hospital, Chengdu 611730, China; caomu.youxin@163.com

**Keywords:** *sorbus pohuashanensis*, yeast extract, biphenyl, phytoalexin

## Abstract

Biphenyls are unique phytoalexins de novo synthesized in plants in response to pathogen attack. These compounds are found in Maloideae, a subfamily of the Rosaceae. The anti-microbial activities of biphenyls have been reported in a number of studies and they appear to represent an important defense strategy against pathogens common in the Maloideae, such as species in *Malus*, *Pyrus*, *Sorbus*, and *Chaenomeles*. Here, cell suspension cultures of *Sorbus pohuashanensis* were established to study biphenyl phytoalexins formation after yeast extract (YE) treatment. An ultra-performance liquid chromatography (UPLC) method coupled with quadrupole time of flight mass spectrometry (Q-TOF-MS) LC−MS/MS was applied to determine the time course of these biphenyl biomarkers accumulation in YE-treated *S. pohuashanensis* suspension cells. The results of quantitative analyses show the content of Noraucuparin, 2′-Hydroxyaucuparin, and their glycosides initially increased, then decreased over time. The Noraucuparin content reached its highest (225.76 μg·g^−1^) at 18 h after treatment, 6 hours earlier than that of Noraucuparin 5-*O*-β-d-glucopyranoside. The content of 2′-Hydroxyaucuparin reached its highest (422.75 μg·g^−1^) at 30 h after treatment, also earlier than that of its glycoside. The understanding of phytoalexin metabolism in this study may provide a basis for improving Maloideae resistance to pathogens.

## 1. Introduction

Phytoalexins, anti-microbial compounds with a low molecular weight that form at local infection sites and other areas, are vital biochemical responses of plants to infections by pathogen, and their formation and accumulation are part of the plant’s resistance mechanisms [1]. Maloideae, including a number of economically important fruit trees, such as apple (*Malus domestica*) and pear (*Pyrus communis*), could de novo synthesize phytoalexins after infection by bacteria and fungi. Even so, there is significant economic loss because yield can be greatly affected by these types of pathogenic infections [2]. The anti-microbial activities of biphenyls and dibenzofurans have been demonstrated in a number of studies [3,4,5,6,7], showing that they can inhibit spore germination, germ-tube development, and mycelial growth. Thus, as one of the anti-pathogen mechanisms, biphenyls and dibenzofurans may enhance the resistance of Maloideae to pathogens.

*Sorbus pohuashanensis*, a deciduous tree, is a species of some medical, edible, garden, and ecological value, with its fruit being used in brewing and medicine. Other species in *Sorbus*, such as *S. aucuparia*, *S. chamaemespilus*, and *S. domestica*, have been reported to produce biphenyls and dibenzofurans after infection of pathogens or treated with elicitors like yeast extract (YE) [8,9,10], but these biphenyls also were aglycone, not linked to glycosides. What’s more, the kind and amount of biphenyls and dibenzofurans vary in different species of Maloideae. For example, a single dibenzofuran was observed in *P. pyraster*, whereas five dibenzofurans were detected in *P. communis* [11]. To date, changes of biphenyls and dibenzofurans in *S. pohuashanensis* after pathogen infection or YE treatment have not yet been reported.

To investigate the formation of biphenyls and their glycosides in *S. pohuashanensis* after YE-treatment, its cell suspension culture was established and treated with YE, then methods of plant metabolomics were used to screen out different phytoalexins, which were later quantitatively analyzed by LC-MS/MS to further explore their changes with time after YE treatment. In this way, we can understand the dynamic changes and metabolic rules of these compounds. The conversion of aglycones to glycosides cyclized by glycosyltransferases (GTs) and the activity of biphenyls and their glycosides are different [3]. In this study, changes in the amount of important phytoalexins and their glycosides over time were dynamically analyzed and the metabolic cycle of part of the phytoalexins was revealed in YE-treated cell cultures of *S. pohuashanensis*, laying a foundation for further study of the resistance mechanism of phytoalexins in Maloideae.

## 2. Results

### 2.1. Establishment of Cell Suspension Cultures

Visible, primary calli formed on root explants after 20 to 25 days, while seeds and stems explants failed to form calli. Primary calli were subcultured several times, and soft and friable calli were preferentially selected and subcultured in liquid MS medium at 2 weeks intervals 3 to 5 times to produce a cell suspension composed mainly of single cells and small cell aggregates.

### 2.2. Study of Growth Properties of Suspension Cells

#### 2.2.1. Growth Curves of Suspension Cells

**Figure 1:**
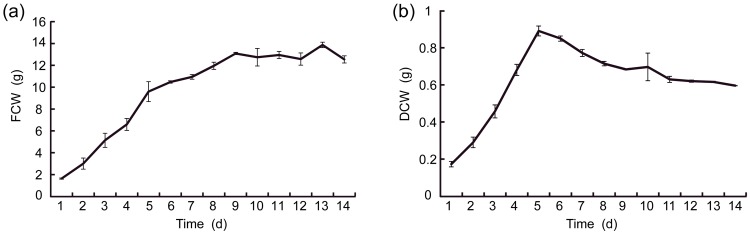
The growth curves of FCW and DCW of *S. pohuashanensis* suspension cell. FCW (**a**) and DCW (**b**) denote respectively the fresh cell weights and the dry cell weights of *S. pohuashanensis* suspension cell.

Growth curves of fresh cell weights (FCW) and dry cell weights (DCW) for *S. pohuashanensis*, as shown in Figure 1, exhibited quick growth without a lag phase at the initial stage of subculture. FCW (Figure 1a) grew rapidly during days 1–5, with growth rate slowing between days 5–9, and reached a plateau between days 9–14; while DCW grew quickly during days 1–5, and slowed over the final 9 days (5–14). DCW reached its peak (0.89 g per flask) on the 5th day after subculture, as shown in Figure 1b, then continued to decrease during the culture, and this was due to the decrease of nutrient intake in cell proliferation caused by nutrient consumption in the later period of the culture.

#### 2.2.2. Fitting of Growth Curve for Suspension Cell

Population quantity of suspension cells, a typical generation overlapping of a single population in a limited space, presents an S curve in a growth cycle [12,13], so the equation of Logistic f(x) = b/(1 + a × exp(−k × x)) was used to fit changes of FCW and DCW with time in a growth cycle, and the results are shown in Table 1.

The degree of fitting of FCW was better than that of DCW, as the R-square described in Table 1, so FCW was used to fit the curve, as following:

f(x) = 13.01/(1 + 11.75 × exp(−0.6503 × x))(1)

#### 2.2.3. Solving Key Point of the Logistic Growth Model

**Figure 2:**
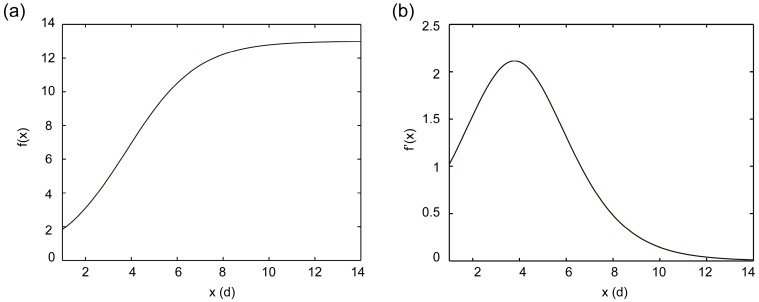
Function curves put out by MATLAB (**a**) represents graph of growth function, while (**b**) graph of its first derivation).

Function (1), plotted using a function of fplot as shown in Figure 2a, suggested that the growth curve of *S. pohuashanensis* suspension cell reached its plateau on the 9th day. The first derivation of Function (1) was obtained and plotted using the function of diff by Matlab 2012b (Mathworks, Natick, MA, USA), as shown in Figure 2b, suggesting growth rate increased before decreasing, reaching its peak on day 4, and then returned to its initial value on day 6. In this study, we chose to add elicitor on day 5 after the subculture was taken.

### 2.3. Analyses of Metabolites in YE-Treated S. pohuashanensis Suspension Cells

#### 2.3.1. Analyses of Time Course of Metabolites Differentially Accumulated in YE-Treated *S. pohuashanensis* Suspension Cells

**Figure 3:**
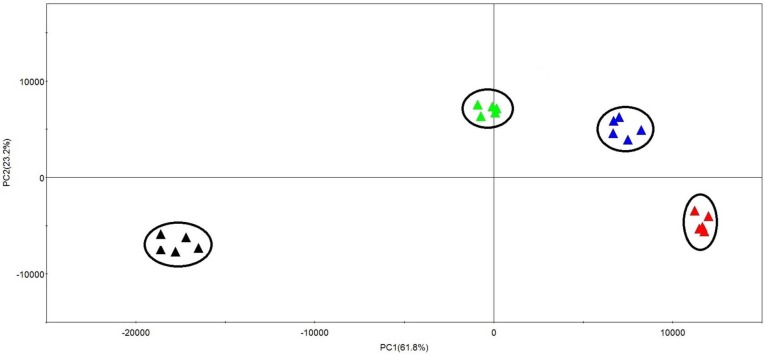
Principal component analysis (PCA) of metabolites from control and treatment groups (YE-treatment after 0 h: ▲; YE-treatment after 12 h: ▲; YE-treatment after 24 h: ▲; YE-treatment after 48 h: ▲) in *S. pohuashanensis* suspension cell analysed by UPLC-QTOF-MS (*n* = 5). Metabolome clusters are located at distinct positions in the two-dimensional space as prescribed by the two vectors of principal component 1 (PC1 = 61.8%) and principal component 2 (PC2 = 23.2%).

A total of 2658 mass signals extracted by Progenesis QI software (version 1.0, Waters, Milford, MA, USA) from the UPLC-MS data set acquired in negative ionization mode were used for Principal Component Analysis (PCA). PCA involves a mathematical procedure for reducing the dimensionality of multidimensional matrices while retaining a large amount of the original information in the data set [14]. Quintuplicate measurements from the same sample were found to be highly reproducible, as the scores of replicate measurements were more or less superimposed. PCA score plots showed that the metabolites in *S. pohuashanensis* suspension cell by YE-treatment after 0 h, 12 h, 24 h and 48 h were clearly clustered into four groups (Figure 3). The samples of 0 h are positioned on the left-hand side of the plot (negative PC1 values), whereas samples of 48 h are located on the far right-hand side (positive PC1 values). Other samples for 12 h and 24 h are spread in between. This result indicates that the metabolites have significant differences between control and treatment groups, and there are substantial differences in metabolites in *S. pohuashanensis* suspension cells after different treatment times.

**Figure 4:**
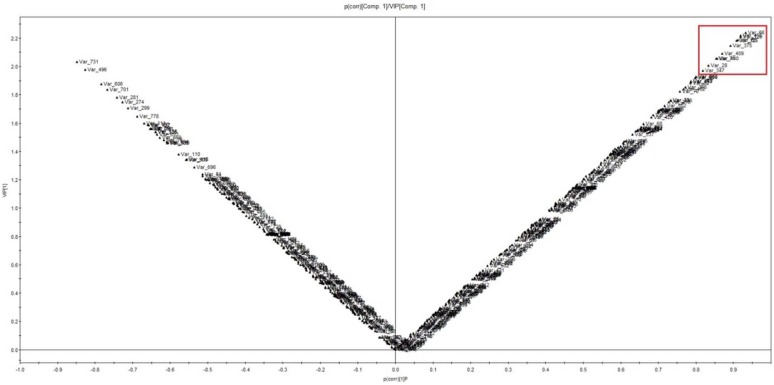
Scatter plot (V-plot) from OPLS-DA analysis for selecting the differential metabolites as potential markers to discriminate the groups of control and YE-treatment after 24 h.

To further study the variance between the different treatment times and to determine potential biomarkers, supervised OPLS-DA (orthogonal partial least squares discriminate analysis) was subsequently used. The R^2^X, R^2^Y, and Q^2^ of this model are 0.645, 0.996, and 0.988, respectively. The permutation result validated the stability and reliability of this OPLS-DA model. Subsequently, relying on the two criteria—VIP (variable importance in the projection) value of OPLS–DA model >1.8 and correlation coefficient [p(corr)/VIP] > 0.8—a total of 18 marker compounds accountable for the different metabolite profiles of control and treatment groups were obtained (Figure 4).

**Figure 5:**
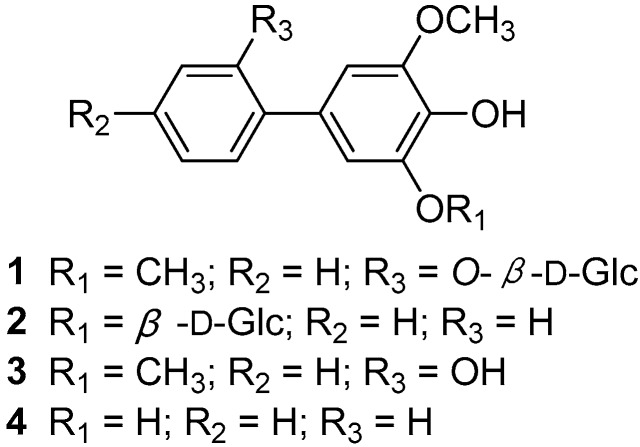
Biphenyl and biphenyl glycosides isolated from YE-treated *S. pohuashanensis* suspension cells.

Through the above research, four MS signals assigned to biphenyl compounds contributed significantly to the classifications of different times, as evident from high-resolution masses *m*/*z* 215.0706 [M − H]^−^, *m*/*z* 245.0814 [M − H]^−^, *m*/*z* 377.1229 [M − H]^−^ and *m*/*z* 407.1331 [M − H]^−^ with predicted molecular formulae of C_13_H_11_O_3_^−^, C_14_H_13_O_4_^−^, C_19_H_11_O_8_^−^ and C_20_H_23_O_9_^−^, respectively. These metabolites could be used as potential markers in different groups of time course of metabolites differentially accumulated in YE-treated *S. pohuashanensis* suspension cells. The makers may be attributed to differences in the activity of key enzymes, and differences in the regulation of metabolic pathways. Furthermore, we separated these compounds from the methanol extract of YE-treated cell cultures of *S. pohuashanensis* by guided LC-MS, and identification structures of these target compounds based on NMR (Figure 5).

#### 2.3.2. Characterization of Biphenyls Compounds

*2′*-*Hydroxyaucuparin 2′-O-β-d-glucopyranoside* (**1**): Yellow amorphous powder (MeOH); UV (MeOH) λ_max_ (log ε) 279 (5.5), 226 (4.6) nm; ESI-MS (negative) *m*/*z* 407 [M − H]^−^, 245[M − H − 162]^−^; HR-ESI-MS (negative) *m*/*z* 407.1331 [M − H]^−^ (Calcd. for C_20_H_23_O_9_, 407.1342); ^1^H-NMR (600 MHz, CD_3_OD): 7.18–7.21 (2H, m, H-4′, 6′), 6.97–7.01 (2H, m, H-3′, 5′), 6.67 (2H, s, H-2, 6), 4.89 (1H, d, *J* = 7.8 Hz, glc-H-l), 3.20–3.75 (6H, m, glc-H-2,3,4,5,6), 3.87 (6H, s, 3, 5-OCH_3_); ^13^C-NMR (150 MHz, CDCl_3_): δ 131.1 (C-1), 106.7 (C-2, 6), 148.8 (C-3, 5), 135.9 (C-4), 131.2 (C-1′), 153.8 (C-2′), 116.5 (C-3′), 128.1 (C-4′), 120.7 (C-5′), 130.3 (C-6′), 56.2 (3, 5-OCH_3_), 101.7 (glc-C-1), 73.7 (glc-C-2), 77.8 (glc-C-3), 70.2 (glc-C-4), 76.3 (glc-C-5), 61.2 (glc-C-6). The spectroscopic properties were in accordance with published data [3,15].

*Noraucuparin 5-O-β-d-glucopyranoside* (**2**): Yellow amorphous powder (MeOH); UV (MeOH) λ_max_ (log ε) 280 (4.0), 229 (4.9) nm; ESI-MS (negative) *m*/*z* 377 [M − H]^−^, 215[M − H − 162]^−^; HR-ESI-MS (negative) *m*/*z* 377.1229 [M − H]^−^ (Calcd. for C_19_H_21_O_8_, 377.1236); ^1^H-NMR (600 MHz, CD_3_OD): δ 7.52 (2H, d, *J* = 7.4 Hz, H-2′, 6′), 7.45 (2H, t, *J* = 7.4 Hz, H-3′, 5′), 7.26 (1H, t, *J* = 7.3 Hz, H-4′), 6.87 (1H, s, H-2), 6.65 (1H, s, H-6), 4.44 (1H, d, *J* = 7.9 Hz, glc-H-l), 3.93 (1H, d, *J* = 11.7 Hz, glc-H-6b), 3.85 (3H, s, 3-OCH_3_), 3.69 (1H, dd, *J* = 11.5, 5.6 Hz, glc-H-6a), 3.50–3.53 (1H, m, H-3), 3.18–3.39 (4H, m, glc-H-2,3,4,5); ^13^C-NMR (150 MHz, CD_3_OD): δ 131.7 (C-1), 102.1 (C-2), 146.5 (C-3), 132.3 (C-4), 149.7 (C-5), 107.5 (C-6), 141.3 (C-1′), 126.9 (C-2′, 6′), 128.5 (C-3′, 5′), 126.4 (C-4′), 56.1 (3-OCH_3_), 101.7 (glc-C-1), 74.7 (glc-C-2), 78.2 (glc-C-3), 71.5 (glc-C-4), 78.1 (glc-C-5), 62.7 (glc-C-6). The spectroscopic properties were in accordance with published data [3,15].

*2′-Hydroxyaucuparin* (**3**): White amorphous powder (CHCl_3_); UV (MeOH) λ_max_ (log ε) 275 (4.2), 230 (4.6) nm; HR-ESI-MS (negative) *m*/*z* 245.0811 [M − H]^−^ (Calcd. for C_14_H_13_O_4_, 245.0814); ^1^H-NMR (600 MHz, CDCl_3_): δ 7.22–7.27 (2H, m, H-4′, 6′), 6.96–6.99 (2H, m, H-3′, 5′), 6.66 (2H, s, H-2, 6), 3.91 (6H, s, 3, 5-OCH_3_); ^13^C-NMR (150 MHz, CDCl_3_): δ 128.2 (C-1), 105.7 (C-2, 6), 147.6 (C-3, 5), 134.5 (C-4), 129.0 (C-1′), 152.5 (C-2′), 115.6 (C-3′), 127.9 (C-4′), 120.7 (C-5′), 130.0 (C-6′), 56.4 (3, 5-OCH_3_). The spectroscopic properties were in accordance with published data [15,16].

*Noraucuparin (4,5-Dihydroxy-3-methoxybiphenyl)* (**4**): White amorphous powder (CHCl_3_); UV (MeOH) λ_max_ (log ε) 275 (4.2), 230 (4.6) nm; HR-ESI-MS (negative) *m/z* 215.0706 [M − H]^−^ (Calcd. for C_13_H_11_O_3_, 215.0708); ^1^H-NMR (600 MHz, CDCl_3_): δ 7.53 (2H, d, *J* = 7.4 Hz, H-2′, 6′), 7.40 (2H, t, *J* = 7.4 Hz, H-3′, 5′), 7.30 (1H, t, *J* = 7.3 Hz, H-4′), 6.86 (1H, s, H-2), 6.70 (1H, s, H-6), 3.93 (3H, s, 3-OCH_3_); ^13^C-NMR (150 MHz, CDCl_3_): δ 132.0 (C-1), 102.3 (C-2), 147.1 (C-3), 133.6 (C-4), 144.1 (C-5), 107.8 (C-6), 141.1 (C-1′), 126.8 (C-2′, 6′), 128.7 (C-3′, 5′), 126.9 (C-4′), 56.2 (3-OCH_3_). The spectroscopic properties were in accordance with published data [16,17].

#### 2.3.3. Calibration Curves, Limit of Detection (LOD), and Limit of Quantitation (LOQ)

To evaluate the linearity, calibration standards of six concentration levels of each analyte were prepared and analyzed. The calibration curve was constructed by plotting the peak area vs. the concentrations. As shown in Table 2, four biphenyl compounds showed good linear regression within the test range. The linear regression analysis results showed that the coefficient estimation of the standard curve was >0.9991. The LOD and LOQ of 4 analytes were estimated to be 0.74–2.47 and 1.91–7.65 ng·mL^−1^, respectively.

#### 2.3.4. Analyses of Biphenyls Accumulated in *S. pohuashanensis* Suspension Cells Treated with Different Concentrations of YE

**Figure 6:**
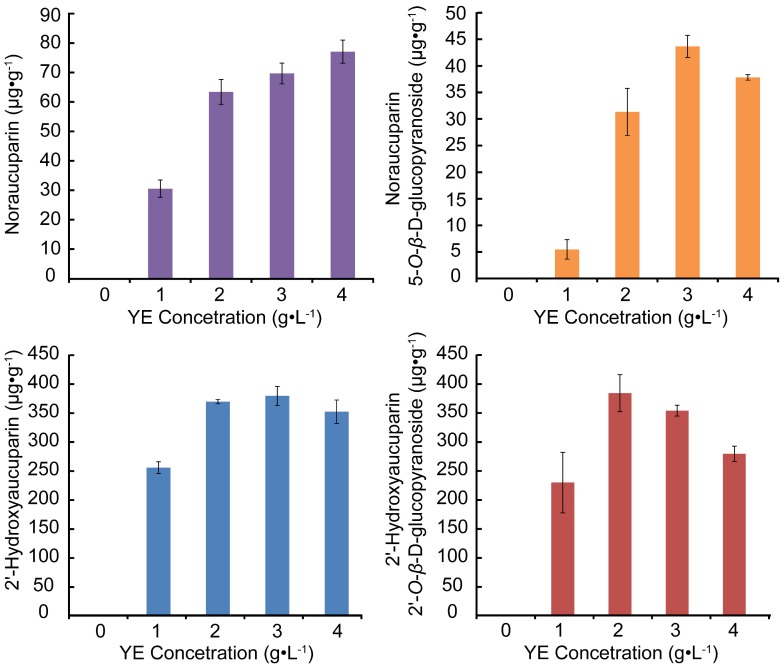
Analyses of contents of four biphenyl biomarkers with different concentrations of YE in *S. pohuashanensis* suspension cells. Values are means of three independent experiments. Bars represent standard errors.

Figure 6 illustrates content changes of the 4 biphenyl biomarkers with different concentrations of YE, and shows contents of Noraucuparin, Noraucuparin 5-*O*-β-d-glucopyranoside, 2′-Hydroxyaucuparin, 2′-Hydroxyaucuparin 2′-*O*-β-d-glucopyranoside increased before decreasing with the increase of YE concentration. Contents of Noraucuparin 5-*O*-β-d-glucopyranoside and 2′-Hydroxyaucuparin reached their highest with YE treatment of 3 g·L^−1^ and contents of 2′-Hydroxyaucuparin 2′-*O*-β-d-glucopyranoside reached highest with 2 g·L^−1^ YE treatment, while content of Noraucuparin increased with the increase of YE concentration, so 3 g·L^−1^ was chosen as the YE concentration for treatment in the following study.

#### 2.3.5. Determination of Time Course of Biphenyls Accumulation in YE-Treated *S. pohuashanensis* Suspension Cells

**Figure 7:**
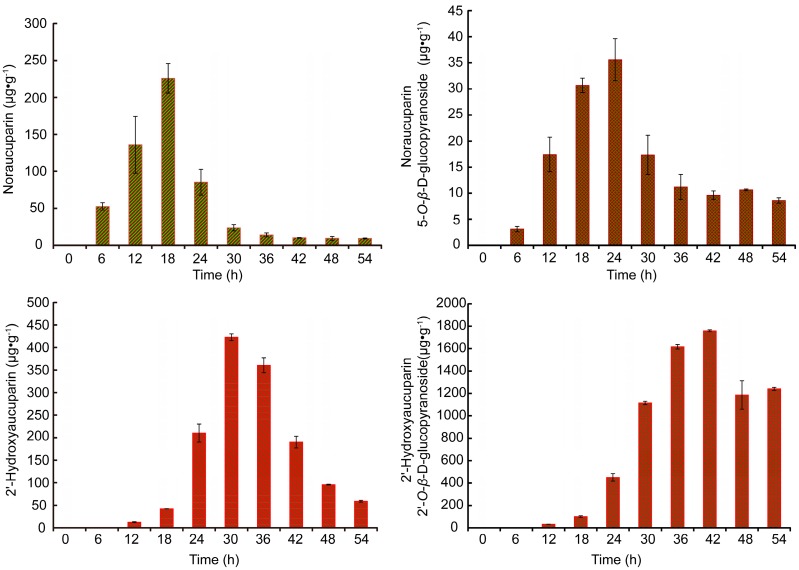
Time course of biphenyl and its glycoside production of *S. pohuashanensis* suspension cells after YE treatment. Values are means of three independent experiments. Bars represent standard errors.

Figure 7 illustrating changes of Noraucuparin and Noraucuparin 5-*O*-β-d-glucopyranoside contents after YE treatment, showed the contents of both increased before decreased with treatment time, and generally remained stable after 36 h. Content of Noraucuparin reached its peak (225.76 μg·g^−1^) at 18 h, while the highest content of Noraucuparin 5-*O*-β-d-glucopyranoside (41.60 μg·g^−1^) was detected at 24 h. Neither of these biphenyls was detectable at 0 h. Otherside, the contents of 2′-Hydroxyaucuparin and its glycosides increased and then decreased over time. Content of 2′-Hydroxyaucuparin reached its peak (422.75 μg·g^−1^) at 30 h, while contents of 2′-Hydroxyaucuparin 2′-*O*-β-d-glucopyranoside reached its peaks (1759.10 μg·g^−1^) at 42 h. Moreover, 2′-Hydroxyaucuparin and 2′-Hydroxyaucuparin 2′-*O*-β-d-glucopyranoside were not detectable at 0 h or 6 h. These results indicate that Noraucuparin and 2′-Hydroxyaucuparin could transfer not only to their downstream mediators but also to their glucosides via glycosyltransferase.

## 3. Discussion

In this study, cell suspension culture of *S. pohuashanensis* was established, and its growth properties studied. This provided us with cells of good dispersibility, small and even cell aggregates, and fast growth for our experiments.

Analysis of the effects of different YE treatment times on the contents of the four biphenyl biomarkers showed that 2′-Hydroxyaucuparin, Noraucuparin, and their glycosides, which are rarely seen in phytoalexins, increased before decreasing over treatment time. The contents of 2′-Hydroxyaucuparin and Noraucuparin reached their respective peaks before their corresponding glycosides, suggesting aglycones formed on YE treatment, then transferred to glycosides by glycosylation with corresponding glycosyltransferase. The gradual decline of 2′-Hydroxyaucuparin after 30 h may be attributed to transforming to a glycoside or dibenzofuran through intramolecular cyclization [11,18]. The considerable decline of 2′-Hydroxyaucuparin 2′-*O*-β-d-glucopyranoside after 42 h may be due to its further transformation to other 2′-Hydroxyaucuparin glycosides which contain more sugar units. Noraucuparin decreased gradually after 18 h, and this may be caused by its transformation to Noraucuparin 5-*O*-β-d-glucopyranoside or other biphenyl by 2′-Hydroxylation or methylation of 3-OH. Interestingly, accumulation trends for 2′-Hydroxyaucuparin, Noraucuparin, and their corresponding glycosides were the same during the treatment, while Noraucuparin reached its peak earlier than 2′-Hydroxyaucuparin, which may due to their different locations in the biphenyl synthesis pathway. Noraucuparin is supposed to be in the upper stream of 2′-Hydroxyaucuparin [11], and the results here verified this hypothesized biphenyl synthesis pathway.

The biphenyl scaffold is formed by biphenyl synthase (BIS), which catalyzes benzoyl-CoA and malonyl-CoA to generate 3,5-Dihydroxybiphenyl [9,19]. In recent studies, the biosynthetic pathway leading from 3,5-Dihydroxybiphenyl to Noraucuparin was clarified at the enzyme level in *S. aucuparia* [18]. The first step is the conversion of 3,5-Dihydroxybiphenyl to 3-Hydroxy-5-methoxy biphenyl catalyzed by *O*-methyltransferase 1 (OMT1). Then, biphenyl 4-hydroxylases (B4H) hydroxylate 3-Hydroxy-5-methoxy biphenyl to yield Noraucuparin. Subsequently, a second OMT activity (OMT2) methylated the 3-hydroxyl group of Noraucuparin to produce Aucuparin. In our study, Noraucuparin could also be catalyzed by glycosyltransferases to generate Noraucuparin 5-*O*-β-d-glucopyranoside, a rare phytoalexin. Similarly, the conversion of 2′-Hydroxyaucuparin to 2′-Hydroxyaucuparin 2′-*O*-β-d-glucopyranoside also exist in YE-treated *S. pohuashanensis* suspension cells.

Previous studies have shown the elicitor-induced accumulations of secondary metabolites in plants are closely associated with reactive oxygen species (ROS), such as superoxide anion (O_2_^−^) and hydrogen peroxide (H_2_O_2_) [20,21,22]. There are many causes leading to a burst of ROS. NADPH oxidases of plasma membrane redox system (PMRS) is one of the main sources of ROS in pathogen infected plant cells [23]. Under the effect of elicitor, NADPH oxidase is rapidly activated and then generates a burst of ROS. Qiu et al. [24] showed that endogenous hydrogen peroxide derived from NADPH oxidase played important roles in the YE-induced activation of biphenyl biosynthesis in cell cultures of *S. aucuparia*.

## 4. Materials and Methods

### 4.1. Plant Materials

Seeds as well as the stems and roots of seedlings from *S. pohuashanensis* were used in this study.

### 4.2. Chemicals and Reagent

HPLC grade methanol and acetonitrile were purchased from the Fisher Chemical Company (Pittsburgh, PA, USA) and formic acid was obtained from Sinopharm Chemical Reagent Co., Ltd. (Shanghai, China). An ultrapure water system (Barnstead Pacific TII; Thermo Scientific, Waltham, MA, USA) was used. The grade 60 silica gel (70–230 mesh, 60 Å Sigma-Aldrich, St. Louis, MO, USA) and silica gel (200–300 mesh, Qingdao Marine Chemical Inc., Qingdao, China).

### 4.3. Construction of Cell Suspension Cultures

Seeds, stems, and roots measuring 3–4 cm were first washed with distilled water several times, then immersed in 75% (*v*/*v*) ethanol for 30 s before surface-disinfection in 0.01% HgCl_2_ solution under continuous agitation for 10 min, and subsequently rinsed 4–5 times with sterile distilled water. Surface water was soaked up by sterilized filter paper, then the plant matter was spread evenly on induction medium, which was later placed in a dark culture room, temperature controlled at 25 °C. Calli with vigorous growth, loose structure, and good dispersion were subcultured on a MS medium in a 250 mL shaker flask, and the flask was incubated in dark conditions at 25 °C on an orbital shaker at 120 r·min^−1^, and cells subcultured 3–5 more times were used as experiment materials.

### 4.4. Growth Property Study of the Suspension Cells

The fresh weight of the cell biomass from each flask was measured. Three culture flasks were taken randomly every day over a 14-day period to get the average weight of the cell biomass. The cells were harvested by filtering the cell suspension cultures through filter paper using a filter funnel connected to a vacuum pump and rinsed with distilled water 3 times to get the FCW. The DCW were recorded after air-drying at 40 °C until constant weights were obtained.

Growth curves of FCW and DCW were fitted using the Logistic model by Matlab 2010b, and recorded weights were computed and put into graphs generated by Microsoft Excel.

### 4.5. Effects of Metabolites in Cell Cultures of S. pohuashanensis Treated at Different Times and with Different Concentrations of YE

Five-day-old cell cultures were treated with YE of 0 (control group), 1, 2, 3 and 4 g·L^−1^ respectively for 24 h. Next, five-day-old cell cultures were treated with YE (3 g·L^−1^), and samples were collected after treatment of 0, 6, 12, 18, 24, 30, 36, 42, 48 and 54 h respectively. This was repeated three times in order to obtain three sets of samples.

### 4.6. Sample Preparation

YE treated cells were collected by filtering the cell suspension cultures through filter paper using a filter funnel connected to a vacuum pump, and dried using vacuum freeze-drying equipment (FDU-1110, EYELA, Tokyo, Japan). 100 mg of dried cell was precisely weighed out in an Eppendorf tube. The samples were sonicated in 1.5 mL of 80% methanol for 30 min followed by centrifugation for 10 min at 12,000 rpm and then the supernatant were filtered with 0.22 μm PTFE membrane filter (Jinteng Technologies, Tianjin, China).

### 4.7. UPLC-Q-TOF-MS Analysis

The metabolite profiling of *S. pohuashanensis* suspension cells was performed on a Waters ACQUITY UPLC I-Class/Xevo in line with a Waters Xevo G2 Q-TOF mass spectrometer controlled with Masslynx (version 4.1, Waters Corporation, Milford, MA, USA). Samples were separated on an Acquity UPLC BEH C_18_ column (2.1 mm × 100 mm, 1.7 μm). The column temperature was maintained at 40 °C. The binary gradient consisted of solvent system A (formic acid/water 0.1:99.9 *v*/*v*) and solvent system B (formic acid/acetonitrile 0.1:99.9 *v*/*v*). The conditions were as follows: 0 min, 5% B; 1 min, 5% B; 6 min, 40% B; 8 min, 52% B; 12 min, 85% B; 15 min, 98% B. The injection volume for all samples was 1 μL and the flow rate was 0.5 mL·min^−1^.

The Waters Xevo G2 Q-TOF mass spectrometer (Waters Corporation, Milford, MA, USA) was run in negative mode. The data were collected for each test sample from 50 Da to 1500 Da. High purity nitrogen (N_2_) was used as nebulizing gas, and ultra-high pure helium (He) as collision gas. Source parameters were as follows: capillary voltage, 2.50 kV; sampling cone voltage, 35.0 V; source offset, 80.0 V; desolvation temperature, 500 °C; cone gas flow, 50 L·h^−1^; and desolvation gas flow, 800 L·h^−1^. To ensure mass accuracy and reproducibility, leucineenkephalin was used as the reference lock mass (*m*/*z* 554.2615) with the LockSpray interface.

### 4.8. Multivariate Data Analysis

Raw profile data (raw) from UPLC-Q-TOF-MS were imported into the Progenesis QI software for removal of isotope peak, alignment and peak-picking. This software approach employs peak alignment, matching, and comparison. After peak extraction and grouping, nonlinear retention time correction of peaks was accomplished in two iterative cycles with descending bandwidth (bw). The resulting data matrix consisting of retention time, *m*/*z* value, and peak height intensity was introduced into SIMCA-P software (version 11.0, Umetrics, Umea, Sweden) for multivariate pattern recognition analysis. All the variables were Pareto (Par)-scaled prior to principal component analysis (PCA) and orthogonal partial least squares-discriminant analysis (OPLS-DA). The PCA results are displayed as a scores plot indicating the scattering of the samples. The biomarkers of the groups of samples were identified by OPLS-DA relying on the two criteria: VIP value and correlation coefficient [p(corr)/VIP].

### 4.9. Extraction and Isolation

Based on biomarkers screening from 4.8, the compounds of biphenyl and biphenyl glycosides were isolated from YE-treated cell cultures of *S. pohuashanensis* guided by LC-MS. YE-treated cell cultures of *S. pohuashanensis* (400.0 g) were extracted three times using the same process described in section 4.6. The filtered extract was combined and condensed at below 40 °C to remove most of methanol. The residue was suspended in H_2_O (about 400 mL) and then successively partitioned with petroleum ether (PE), EtOAc, and BuOH. The EtOAc extract was freeze-dried and yielded 10.5 g of yellow powder.

The yellow powder (10.5 g) was subjected to column chromatography over silica gel and eluted with CH_2_Cl_2_-MeOH (from 9:1 to 3:1, *v*/*v*) to afford fractions I-V. The Fraction I was further chromatographed on silica gel 60 with a stepwise gradient of CH_2_Cl_2_–acetone (100:0, 98:2, 95:5, 90:10, 80:20, 0:100) to obtain 6 fractions (Fractions 1.1→1.6). Fr.1.3 was subjected to be repeated CC over silica gel 60 (stepwise, hexane–ethyl acetate from 2:1 to 1:1) to afford **3** (6 mg) and **4** (8 mg). Preparative HPLC was performed on Shimadzu LC-10A instrument equipped with a SPD-M20A detector (Shimadzu Corporation, Kyoto, Japan) using YMC-Pack ODS-A columns (20 mm × 250 mm, 5.0 μm). Fraction IV was chromatographed over silica gel 60 eluting with CHCl_3_–MeOH–EtOAc–H_2_O (5:3:3:1, *v*/*v*, lower layer) to afford Fraction IVa, Fraction V with CHCl_3_–MeOH–EtOAc–H_2_O (3:2:2:1, *v*/*v*, lower layer) to afford Fraction Va. They were further purified by preparative HPLC using acetonitrile and water as the mobile phase (flow rate 8.0 mL·min^−1^) and the eluate was monitored at 276 nm. Compound **2** (3 mg) was obtained from Fraction IVa with gradient elution (23%–25% acetonitrile from 25–35 min), and Compound **1** (10 mg) was obtained from Fraction Va by using 21.5% acetonitrile.

### 4.10. LC-MS/MS Analyses

Chromatographic analyses were performed on an Acquity UPLC I-Class system (Waters). UPLC separation was carried out on Acquity UPLC BEH C_18_ column (2.1 mm × 100 mm, 1.7 μm) thermostated at 40 °C. The binary gradient consisted of solvent system A (formic acid/water 0.1:99.9 *v*/*v*) and solvent system B (formic acid/acetonitrile 0.1:99.9 *v*/*v*). The conditions were as follows: 0 min, 10% B; 4 min, 40% B; 5 min, 52% B. The injection volume for all samples was 1 μL and the flow rate was 0.6 mL·min^−1^.

Tandem mass spectrometry was performed on the QTRAP 6500 system (AB SCIEX, Los Angeles, CA, USA) for characterization equipped with electrospray ionization (ESI) source (AB SCIEX). MS analysis was carried out in negative ionization mode (ESI) by monitoring the protonated molecular ions at the following operating conditions: ion source voltage of −4500 V; entrance potential (EP) of −10 V; nebulizer gas (Gas 1) of 55 psi; heater gas (Gas 2) of 55 psi; curtain gas of 30 psi and turbo spray temperature (TEM) of 550 °C. MS parameters were manually optimized as shown in Table 3. Quantification was determined using multiple reaction-monitoring (MRM) modes for the above transitions. Data were acquired using Analyst software 1.6.2. (AB SCIEX, Los Angeles, CA, USA) and analyzed using MultiQuant Software 3.0 (AB SCIEX, Los Angeles, CA, USA).

## 5. Conclusions

Herein, Noraucuparin and Noraucuparin 5-*O*-β-d-glucopyranoside were detected at 6 h after YE treatment. These showed that de novo biosynthesis of biphenyl phytoalexins was rapidly induced by YE in *S. pohuashanensis* cell cultures. Through analyzing the changes in the four main phytoalexins in terms of different contents with treatment time, we found that Noraucuparin could transfer to Noraucuparin 5-*O*-β-d-glucopyranoside by glycosylation, while 2′-Hydroxyaucuparin could also transfer to a corresponding glucoside, indicating that Noraucuparin and 2′-Hydroxyaucuparin could transfer not only to their downstream mediators but also to their glucosides via glycosyltransferase.

## Figures and Tables

**Table 1:** Fitting of growth function parameters for *S. pohuashanensis* suspension cell.

	Parameters			
	a	b	k	R-Square
FCW	11.75	13.01	0.6503	0.9881
DCW	20.84	0.7068	1.367	0.7839

**Table 2:** Regression equation, correlation coefficients, linearity ranges, and limits of detection (LOD) and quantitation (LOQ) of the biphenyl compounds.

Analytes	Calibration Curves	*r*	Linear Range (μg/mL)	LOD (ng/mL)	LOQ (ng/mL)
Noraucuparin	*y* = 5854.12*x* − 2346.20	0.9993	0.510–25.500	1.01	2.89
Noraucuparin 5-*O*-β-d-glucopyranoside	*y* = 6654.89*x* − 1009.62	0.9991	0.220–11.000	0.74	1.91
2′-Hydroxyaucuparin	*y* = 5382.90*x* − 2.39 × 10^4^	0.9995	5.200–104.000	2.47	7.65
2′-Hydroxyaucuparin 2′-*O*-β-d-glucopyranoside	*y* = 4365.74*x* − 3.72 × 10^4^	0.9993	10.500–210.000	1.38	3.67

**Table 3:** MS analysis on parameters of nine compounds.

Analytes	MRM Transitions (*m*/*z*)	Declustering Potential (DP)/V	Collision Energy (CE)/V	Collision Cell Exit Potential (CXP)/V
Noraucuparin	214.9→199.9	−91	−24	−23
Noraucuparin 5-*O*-β-d-glucopyranoside	376.9→214.9	−95	−22	−12
2′-Hydroxyaucuparin	245.1→214.9	−75	−27	−15
2′-Hydroxyaucuparin 2′-*O*-β-d-glucopyranoside	406.9→244.9	−94	−21	−24
